# The Effects of a 12-Week-Long Sand Exercise Training Program on Neuromechanical and Functional Parameters in Type II Diabetic Patients with Neuropathy

**DOI:** 10.3390/ijerph20075413

**Published:** 2023-04-05

**Authors:** Judit Prókai, Zsolt Murlasits, Miklós Bánhidi, László Csóka, Viktória Gréci, Tamás Atlasz, Márk Váczi

**Affiliations:** 1 Doctoral School of Health Sciences, Faculty of Health Sciences, University of Pécs, 7621 Pécs, Hungary; 2 Institute of Sport Sciences and Physical Education, Faculty of Sciences, University of Pécs, 7624 Pécs, Hungary; 3 Faculty of Health and Sport Sciences, University of Győr, 9026 Győr, Hungary; 4 Department of Marketing and Tourism, Faculty of Business and Economics, University of Pécs, 7622 Pécs, Hungary; 5 Department of Neurology, Medical School, University of Pécs, 7624 Pécs, Hungary; 6 Gyógypont Rehabilitation, 7623 Pécs, Hungary

**Keywords:** physical therapy, unstable surface, balance, gait, EMG, co-activation

## Abstract

Studies have proven the effectiveness of different weight-bearing exercise interventions for diabetic patients with neuropathy; however, several adverse effects were reported using solid surfaces. Thus, in the present study, we investigated the effects of a novel sand exercise training intervention on biomechanical and functional parameters in seven diabetic patients (age = 62.7 ± 9.7 years) with neuropathy. Patients underwent a 12-week sand exercise training program, using strengthening, stretching, balance, and gait exercises. They were tested for ankle plantar- and dorsiflexion peak torque, active range of motion (ROM), timed up and go (TUG), and bilateral static balance. EMG activity of tibialis anterior (TA), gastrocnemius medialis (GM), and lateralis (GL) muscles were measured during unilateral isometric contraction in plantar- and dorsiflexion. In the intervention period, plantarflexion peak torque improved significantly (*p* = 0.033), while dorsiflexion torque remained unchanged. Plantar- and dorsiflexion ROM increased (*p* = 0.032) and (*p* = 0.021), respectively. EMG activity of GM (*p* = 0.005) and GL (*p* = 0.002) measured during dorsiflexion and postural sway in the balance test, as well as time to complete the TUG test, decreased significantly (*p* = 0.021) and (*p* = 0.002), respectively. No adverse effect was reported during the intervention period. We concluded that sand exercise training can be a safe and effective method to improve plantarflexion strength, ankle flexibility, and balance, which is reflected in better gait function in patients with diabetic peripheral neuropathy (DPN).

## 1. Introduction

Today, more than 500 million people are affected by diabetes worldwide [1]. The different types of neuropathies affect approximately half of the patients [2,3,4,5]. Overall loss of foot function and their related neuromechanical properties are the most pronounced manifestations of diabetic peripheral neuropathy (DPN) [6]. Dysfunction of the muscles of the lower leg, such as reduced joint mobility, is observed in patients with DPN [7,8,9]. These limitations caused by DPN lead to postural instability and changes in walking kinematics [10,11,12].

Despite the aforementioned motor disabilities in DPN patients, the number of research studies conducted with pharmaceutical treatments far exceeds the number of those using physical activity. Results from studies investigating the effects of resistance exercise training are inconsistent. A research study reported no changes after strength training in dorsi- and plantarflexion peak torque after 12 weeks [13]. Furthermore, despite that a 10-week exercise training improved thigh and gluteal muscle strength, the foot muscles, that are mostly exposed to diabetes, were not targeted in this study [14]. A 12-week training intervention using strengthening exercises did not improve the measures of dorsiflexion, only plantarflexion [10].

The increased stiffness of the shank musculature in DPN may abnormally affect agonist-antagonist crosstalk. Measuring antagonist muscle co-activation is a common way to test exercise-induced neural adaptation and agonist contraction efficiency; however, in the case of DPN, such information is unavailable. Furthermore, in DPN patients, ankle-joint dorsi flexor activation is prolonged compared to non-neuropathic diabetic or healthy individuals [15]. After an 8-week sensorimotor and gait intervention, the activation of tibialis anterior (TA) and gastrocnemius medialis (GM) decreased in standing, and increased during treadmill walking; however, the duration of each session was 80 min 3×/week, which might not be feasible for everyone [16]. The above data suggest that exercise intervention studies should extensively investigate and monitor the neuromechanical mechanisms that explain either improvement or no improvement in motor function.

Studies targeting improvements in ankle range of motion (ROM) found inconsistent results, showing no change after home-based stretching [8], but a favorable change after a supervised program [17]. The case was the same in studies investigating the effects of balance training; there was no improvement in balance after a home-based intervention [18], but supervised balance training programs improved balance [19] or developed transfer in walking ability [20]. The data above suggest that exercise supervision is a key element in developing both flexibility and balance in patients with DPN.

Along with the favorable effects of resistance and balance exercises that are demonstrated in some investigations, the adverse effects to DPN patients should be considered. In the presence of limited joint mobility, for example, the foot is unable to provide a shock-absorbing mechanism and may lose its ability to maintain normal plantar pressures [21]. This suggests that the exercise surface should be considered. In healthy individuals, exercises on solid surfaces were shown to induce greater muscle acidification and micro-damage compared to other surfaces (e.g., sand, grass, sponge mats) [22,23]. The American Diabetes Association (ADA) issued precautions for exercise training because certain physical activities are not safe for individuals with neuropathy [24]. Acute injuries, such as calf strain during treadmill walking [25], or the development of pain in the Achilles tendon after exercise on a solid surface [20], as well as muscle soreness [10], have been reported in DPN patients. To avoid such adverse effects, exercising on a soft surface could be an alternative for DPN patients. A soft surface such as sand might reduce the load on the tendons both during the landing and the push-off phase [26]. According to ADA, a combination of strength and balance exercises is recommended for DPN patients [27], and sand can provide both resistance and an unstable surface. Due to the force-absorbing ability of sand and the slippage of the sole, the increased contraction time in the concentric phase allows the contracting muscles of the foot to become more active [28]. As the grains of sand slide over each other, applying more body weight to certain parts of the sole creates angular displacement in the opposite direction in the ankle joint, namely a stretching effect; however, this has not yet been proven. Previous studies have shown improvements in balance in patients with chronic ankle instability [29], balance capacity, proprioceptive sensation, and muscle activation in elderly women after training on sandy surfaces [30]. Therefore, exercising in sand may allow favorable strength, balance, and functional changes in DPN patients, while reducing injury risks; however, this hypothesis has not been investigated.

Taken together, because of potential issues of exercising on solid surfaces, we need to develop safer exercise modalities that improve foot function while avoiding the adverse effects in these patients. In addition to the reduced injury risks, sand exercises could potentially improve foot function by combined stimuli of resistance, balancing, and muscle stretching. Thus, in the present study, we investigated the effects of a 12-week sand exercise training program on foot function in DPN patients. We approached this question by measuring ankle ROM and walking ability, and by using quantitative dynamometry and electromyography to measure the neuromechanical properties of ankle plantar- and dorsiflexor muscles. We hypothesized that diabetic patients participating in our training program would show significant improvements in these parameters.

## 2. Materials and Methods

### 2.1. Participants

Seven (female, n = 4, male, n = 3) DPN patients with neuropathy (age = 62.7 ± 9.7 years; body mass = 96.04 ± 25.5 kg; height = 172.57 ± 9.5 cm) participated in the study. Patient information on health status and physical activity was obtained by verbal questioning. The inclusion criteria were diagnosed diabetes according to WHO standards for at least seven years, the ability to walk alone, an inactive lifestyle, and no ulcers in the past six months. Exclusion criteria were partial lower limb amputation, participation in regular physical activity in any form, severe liver failure, severe heart failure (NYHA III-IV), active autoimmune diseases, infection, pregnancy, or breastfeeding. Subjects provided written informed consent according to the Declaration of Helsinki after receiving both a verbal and a written explanation of the experimental protocol and its potential risks. The University Ethics Committee of the University of Pécs approved the protocol (approval number: 5812.-PTE2016).

### 2.2. Experimental Protocol

**Figure 1:**
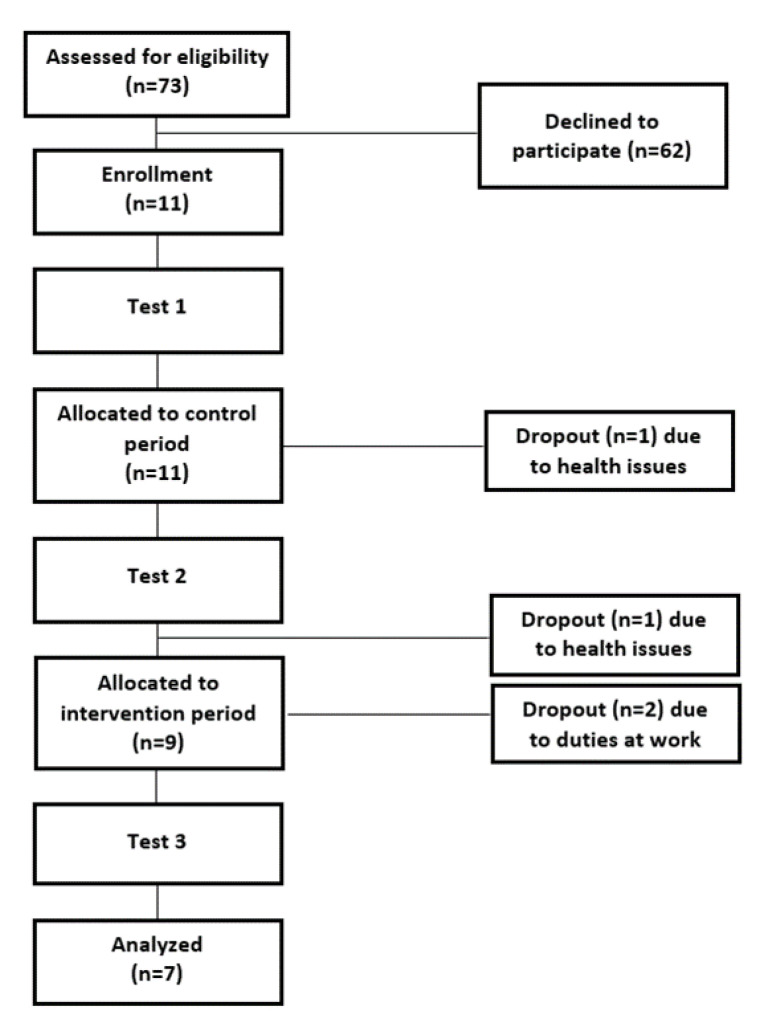
Flowchart of the protocol steps.

This study included a 12-week control period where participants continued their usual medical care with no additional exercises: pharmacological treatment for diabetes and foot care instructions, followed by a 12-week intervention period with sand training. Body composition, ankle plantar- and dorsiflexion torque and EMG activity, ankle ROM, balance and timed up and go (TUG) test time were evaluated at three test sessions: before the control period (Test 1), at the end of the control period which was the beginning of the intervention period (Test 2), and at the end of the intervention period (Test 3). Participants attended one familiarization session before the first laboratory test session to get acquainted with the test tasks (Figure 1). In every test session, participants warmed up by riding a cycle ergometer for 5 min at a self-selected speed.

### 2.3. Body Composition

Body composition analysis was conducted with a bioimpedance analyzer (InBody 720, Biospace, Cerritos, CA, USA). Body mass, skeletal muscle mass, and body fat percentage were measured.

### 2.4. Maximal Voluntary Isometric Contraction (MVIC) Testing

**Figure 2:**
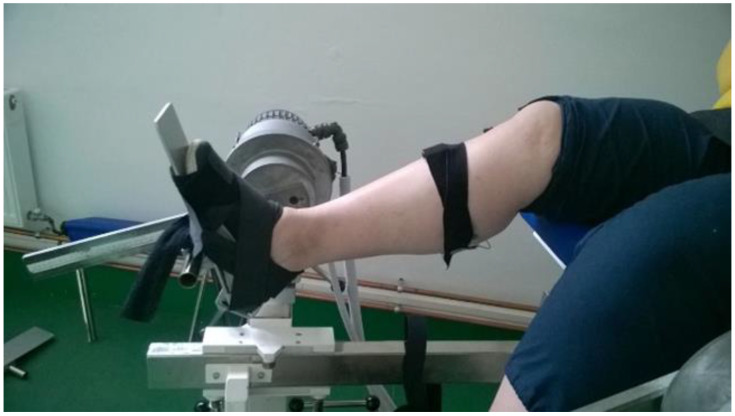
Foot position for testing MVIC plantar- and dorsiflexion on Multicont II isokinetic device.

Unilateral isometric ankle plantar- and dorsiflexion MVICs were performed using Multicont II isokinetic device (Mediagnost, Budapest and Mechatronic Ltd., Szeged, Hungary, sampling frequency: 1000 Hz). Only the dominant leg was tested for every participant, which was decided by asking them to kick a ball [31]. Before any testing, two submaximal warm-up trials were executed. Participants were seated on the dynamometer’s padded seat and performed full effort trials of ankle plantar- and dorsiflexion at 90° of neutral ankle joint position. There were three trials in each MVIC, and a two-minute rest was allowed between trials (Figure 2). The peak torque was determined and used for data analysis. Ankle plantar- and dorsiflexion torque ratios (Mpf/Mdf) were also determined.

### 2.5. Surface Electromyography

**Figure 3:**
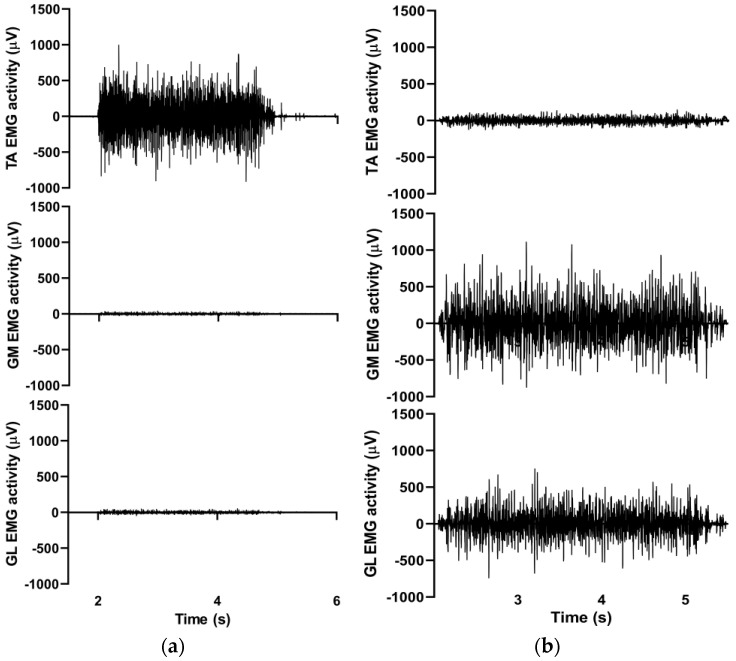
Representative raw EMG signals (RMS) obtained from TA, GM, and GL muscles during plantarflexion (**a**) and dorsiflexion (**b**).

EMG data were collected telemetrically during all MVICs. The skin was carefully prepared by shaving and cleansing with alcohol. Dual Ag/AgCl surface electrodes (Noraxon, Scottsdale, AZ, USA) were positioned on the tibialis anterior (TA), gastrocnemius medialis (GM), and gastrocnemius lateralis (GL) muscles according to the SENIAM recommendations (www.seniam.org). EMG signals were collected (Noraxon, Scottsdale, AZ, USA, sampling frequency: 2000 Hz) and the raw data were processed with the root mean square (RMS) technique, using a 50 ms moving window. Then, the peak EMG values were determined for every MVIC trial. (Figure 3).

### 2.6. Range of Motion (ROM) Testing

The participants were sitting on a mat with their knees extended on the floor and with their torsos leaning against a wall. ROM was measured using a universal goniometer with arms that were 30 cm long. The protractor portion was divided into one-degree increments. A small scale on one of the arms made it possible to obtain measurements to the nearest degree. Unilateral ankle static active range of motion in the sagittal plane was measured by performing a maximal flexion in both plantar- and dorsiflexion, and holding it for 3 s by the patients. Only the dominant leg was tested for every participant.

### 2.7. Balance Testing

A stabilometer (STRUKTURA Instruments Ltd., Tura, Hungary) was used to measure balance during a bilateral stance maintained for 30 s. Participants stood on the stabilometer barefoot with both feet, with hands placed on the hips, and eyes were kept open and fixed on a cursor appearing in the middle of the screen [32]. Visual feedback on actual postural position was continuously provided by the cursor on the screen. Patients were required to keep the cursor as close to the marked screen center as possible during trials. Three trials were performed, with a one-minute recovery between trials. The instrument aggregated the postural sway during the test and evaluated the performance between 0 and 100 points (100 points = perfect balance, no postural sway). The average value of the three trials was calculated and used for statistical analyses.

### 2.8. Timed up and Go (TUG) Test

The TUG test is a valid and reliable method to evaluate walking function in diseased and elderly individuals [33,34]. Patients were seated on a standard chair with a height of 46 cm, with their backs against the seat-back. At the “go” signal, patients got up, walked a 3 m distance, turned around a cone and returned to the chair to sit down again. It was required to walk as fast as possible in a comfortable and safe way. A stopwatch was used to measure the time to complete the test, in seconds. Before any testing, a warm-up trial was performed.

### 2.9. Sand Exercise Training

The training sessions took place three times per week for 12 weeks in a heated, 4 × 5 m sandbox. The participants worked in groups, barefoot in the sand. Each session contained a low-intensity dynamic warm-up (5 min), and lower leg- and ankle-specific exercises (25 min). The duration of the sessions with the number, complexity, and repetition of the exercises increased progressively every four weeks. The training targeted the development in strength and ROM of the muscles around the ankle joint and balance. The training plan was approved by a physiotherapist. The complete description of the exercises can be found in Supplementary Files (Table S1). According to the attendance record, the participation was above 80% for all participants. The drop-out of four patients during the program was due to occupational or other health reasons not related to the study. Patients were asked to report any injury or unusual pain during the intervention period. There were no adverse effects reported during the 12-week sand training.

### 2.10. Statistical Analyses

Group means and standard deviations were computed for all measured and calculated variables. The Shapiro–Wilk tests revealed that all variables were normally distributed, except the anthropometric parameters. In order to detect and measure the differences between Test 1 and Test 2, which was the control period, and Test 2 and Test 3, which was the intervention period, paired sampled t-tests with the appropriate Bonferroni adjustments were performed. Therefore, the corrected *p* value was obtained by dividing the original *p* value (0.1) by the number of comparisons, which was two in every case in this study, finally resulting in *p* = 0.05. In case of not normally distributed data, we used Wilcoxon signed rank test. Cohen’s d values were computed to determine actual effect sizes.

## 3. Results

None of the patients reported any injury or unusual pain during the intervention period. There were no significant changes in any of the measured variables from Test 1 to Test 2 (Table 1). The changes from Test 2 to Test 3 (Table 1) are as follows: Plantar flexor peak torque increased significantly by 42% (*p* = 0.033, ES = 0.603), while dorsiflexion peak torque remained unchanged. During plantarflexion, all EMG data remained unchanged. During dorsiflexion, the EMG activity of GL, GM, and TA decreased significantly, by 30% (*p* = 0.002, ES = −1.414), by 37% (*p* = 0.005, ES = −1.167), and by 23% (*p* = 0.002, ES = −0.697), respectively. Ankle ROM increased significantly both in the plantar- and the dorsiflexion direction, by 18% (*p* = 0.032, ES = 0.504) and 140% (*p* = 0.021, ES = 0.636), respectively.

Postural sway was significantly reduced by 16% (*p* = 0.021, ES = −0.537). The time to complete the TUG test decreased significantly by 18% (*p* = 0.002, ES = −0.667). Body mass, body fat, and muscle mass did not change statistically.

## 4. Discussion

The main findings of this investigation are that a 12-week-long exercise intervention using sand surface: (i) selectively improved plantar flexor peak torque without improvement in dorsiflexion peak torque; (ii) improved balance, walking speed, and ankle sagittal ROM both in plantar- and dorsiflexion; and (iii) decreased EMG activity of GM and GL during dorsiflexion in DPN patients. 

During the 12-week program, body mass and body fat percentage did not change in our patients. This is not surprising, because our training program did not target body composition as the goal was to improve the functional performance of the lower limb. Patients were instructed to maintain their eating habits throughout the entire protocol, and the exercise program was low-volume and low-intensity; therefore, negative energy balance, which would influence body composition [35], was unlikely.

Ankle plantarflexion strength improved by 42%, which is in line with the results of an exercise-based intervention research where patients participated in a combined strength and balance training for 60 min/session, twice a week for 12 weeks. The sessions contained balance and walking tasks alternated with functional, strength, and endurance exercises. The intensity progressively increased by changing the surface from stable to unstable [36]. Regardless of one’s health status, in short-term exercise interventions, the origin of improvement is most likely neural [37]. However, we found no increases in EMG activity in any plantar flexor muscles during plantarflexion MVIC task, indicating that intramuscular coordination was not responsible for the strength gain. Additionally, we found no change in TA activity during the plantarflexion MVIC task after the 12-week exercise, suggesting that plantarflexion strength improvement was also independent from antagonist co-activation. However, the results showed an increasing trend for both GM and GL EMG results after 12 weeks of training, suggesting that the duration of the intervention was too short to make a significant difference. A possible explanation for plantar flexor strength gain is that, besides the sagittal plane exercises, frontal plane exercises perhaps enhanced inter-muscular coordination, involving the evertor/invertor muscles as synergists into plantarflexion function. For example, patients lifted sand and placed it to the side, or drew palm trees with dynamic movements shifting from plantar- to dorsiflexion while whipping the sand according to the proper pattern. Venkataraman et al. [38] reported an improvement in dorsiflexion strength after an 8-week (3 times per session) training intervention where they used not only plantar- and dorsiflexion, but also eversion and inversion exercises for the ankle joint. However, we did not measure the contribution of synergist muscle activation during either a plantar- or dorsiflexion, which is an important limitation.

In our study, dorsiflexion peak torque did not change in the intervention period. Tuttle et al. [13] reported similar results after a 12-week training consisting functional strengthening exercises using only one bodyweight dorsiflexion exercise (two-leg heel stands). In contrast, another research study [10] found significant improvement in dorsiflexion after a combined training program of 40–60 min per session conducted twice a week for 12 weeks. Besides stretching and walking, shank muscles were trained with the use of elastic bands, suggesting perhaps that dorsiflexion can only be improved by externally controlled resistance exercises. Yet, it must be mentioned that more than 40% of the participants were physically active in that study and, additionally, the testing procedures and data were poorly documented. The lack of dorsiflexion strength gain in our patients can also be attributed to the fact that torque was measured only in a 90 degree angle (neutral) position. We found a 140% improvement in dorsiflexion ROM, suggesting that optimal muscle length (i.e., the length at which the muscle is able to produce the highest force) might have changed, and perhaps dorsiflexion strength developed at smaller joint angles toward dorsiflexion. Interestingly, in our study, TA activity during dorsiflexion MVIC reduced without any deterioration in dorsiflexion torque. This suggests perhaps improved cellular calcium release, enabling the muscle to produce the same torque with less activation. Yet, why patients used such an activation strategy, remains unclear. We also found decreased EMG activity in GM and GL during the dorsiflexion MVIC test. Therefore, it seems that the present exercise intervention improved not only plantar flexor strength but also its ability to relax when the opposing muscle was put to work. The reduced antagonist co-activation alone, however, was not enough to lead to agonist (dorsiflexion) strength gain in our case. Altogether, though in our study we targeted dorsiflexion strength by applying dorsiflexion exercises against sand resistance, these stimuli do not seem sufficient to increase dorsiflexion strength. We also know that the inability to stand on heels is more frequent than the inability to stand on toes in neuropathic patients [39]. This may suggest that sand made it even more difficult to do the exercises properly in the direction of dorsiflexion (when patients were requested to stand on heel). A poor baseline ankle ROM could have also prevented dorsiflexor strength gain. At the beginning of the intervention, patients’ ankle ROM in the direction of dorsiflexion was limited, affording only little movement in the ankle joint, thus reducing the strengthening effect. More emphasis should be put on the intensity of the exercises by applying the right external load.

In the present study, ankle ROM increased both in plantar- and dorsiflexion directions. A research study found similar results, where ankle dorsiflexion improved after a 12-week foot-ankle training carried out on a solid surface [17]. However, this training consisted a 2 + 2 times per week supervised and digitally supervised home-based program, 50 min per session. In contrast, a home-based combined training involving a stretching part showed no difference in joint mobility in the intervention group, compared to the control group [8]. In the stretching part of the training, only one exercise was applied for improving ROM of the ankle joint. Thus, failure can be attributed to insufficient exercise stimulus and the home-based format of the intervention. We applied exercises where the feet were forced to move along the whole sagittal ROM of the ankle joint. During pulling and pushing the feet forward and backward with the dorsal and plantar surface of the feet, respectively, the resistance of sand created a passive stretching effect, which could help to improve the range of motion of the ankle joint. In addition, in every exercise, the texture of the sand itself created a constantly unstable surface which can move the feet out from their natural position. Thus, when the sand is slipping out from under the feet, according to the direction of the slippage, it will make the ankle joint tilt to the opposite direction. Since there is a lot of friction among the grains of sand, the movement of the sand will be of medium speed, so the rate of change in the angular position of the joints will also be moderate. Slow or medium speed dynamic stretching exercises can avoid triggering the unwanted stretch reflex [40]. Additionally, during the slippage, the whole foot is supported by the sand, which makes the movement more controllable. Thus, this way of stretching can be safe and effective.

Reduced muscle contractility and ROM caused by DPN lead to postural instability and reduced walking kinematics [10]. In the present study, both bilateral static postural stability and walking speed improved after the intervention period. Our results are in line with the results of other studies after using supervised balance exercises [41,42]. Kruse et al. [18] showed no improvement in balance measurements in DPN patients after eight sessions of instructed balance training in the first 3 months, following home-based balance exercises for 9 months. Although we did not investigate the effectiveness of the same training on a solid surface, previous research studies have shown that balance training on an unstable surface is effective in developing balancing abilities in older adults and that this development occurs earlier than the results achieved by balance exercises on a stable surface. This suggests that balance exercises on unstable surfaces allow a faster development in balance [43]. During every strengthening and stretching exercise carried out on one leg, the other leg served as the support leg and maintained balance, so the working leg was able to execute the actual task. It means that every unilateral exercise trains both legs, but differently: a stretching and/or strengthening effect on one leg, and balance development effect on the contralateral leg.

Stride length is shorter and stance time is longer in DPN compared to diabetes subjects and healthy controls [11], resulting in reduced walking speed in DPN [12]. The time needed to complete the TUG test reduced after our 12-week intervention period. This is in agreement with other studies using stretching, strengthening, balance, endurance, and walking exercises for 8–12 weeks [20,38]. We used specific gait exercises from week 5 of the 12-week training: patients walked forward with normal and longer step length, as well as on tiptoe and heel. We also emphasized strengthening and stretching the muscles around the ankle joint, using exercises in every plane and direction, similar to another study [38]. It is reported that increased ankle strength and joint mobility may contribute to the development of gait velocity [20]. This suggests that the simultaneous strengthening and mobilization of the ankle joint plays a significant role in achieving proper gait function.

The present study has several limitations that warrant consideration. First, we could recruit only a small cohort of DPN patients because of limited volunteering. Second, the contribution of the synergist muscles to either plantar- or dorsiflexion was not evaluated. Finally, we were unable to realize dynamic test contractions due to the dramatically limited baseline ROM in our patients, still, future studies should also use these tests to evaluate angle-specific torque gains in the ankle joint in patients with less severe ROM deficit.

## 5. Conclusions

In conclusion, the present study provides evidence that a 12-week-long sand training intervention that has a multifaceted effect would be a useful therapy in case of DPN, by improving plantar flexor peak torque, balance, walking speed, and ankle sagittal ROM both in plantar- and dorsiflexion without the occurrence of any adverse events.

## Figures and Tables

**Table 1:** The effects of the 12-week sand training on mechanical and function variables and body composition in DPN patients.

Variables		Control Period		Intervention Period		
		Test 1	Test 2	Test 3	∆% ^#^	sig.
Plantarflexion MVIC test						
Torque	(Nm)	51 ± 32	43 ± 22	61 ± 36	18 (42)	*
EMG_GL_	(µV)	99 ± 71	69 ± 69	82 ± 76	13 (19)	
EMG_GM_	(µV)	104 ± 79	87 ± 109	106 ± 119	19 (22)	
EMG_TA_	(µV)	26 ± 9	21 ± 11	20 ± 9	−1 (5)	
EMG_GL/TA_	(ratio)	3 ± 3	3 ± 2	4 ± 3	1 (33)	
EMG_GM/TA_	(ratio)	4 ± 3	4 ± 4	5 ± 5	1 (25)	
Dorsiflexion MVIC test						
Torque	(Nm)	34 ± 21	35 ± 15	33 ± 15	−2 (6)	
EMG_GL_	(µV)	18 ± 9	17 ± 4	12 ± 3	−5 (30)	**
EMG_GM_	(µV)	16 ± 10	19 ± 6	12 ± 6	−7 (37)	**
EMG_TA_	(µV)	285 ± 130	297 ± 99	228 ± 99	−69 (23)	**
EMG_TA/GL_	(ratio)	18 ± 10	18 ± 6	19 ± 9	1 (6)	
EMG_TA/GM_	(ratio)	22 ± 13	17 ± 6	21 ± 12	4 (24)	
Functional measurements						
Postural sway	(points)	43 ± 17	43 ± 14	50 ± 12	7 (16)	*
ROM_PF_	(degree)	51 ± 20	50 ± 21	59 ± 14	9 (18)	*
ROM_DF_	(degree)	−6 ± 9	−5 ± 11	−12 ± 11	7 (140)	*
TUG	(s)	11 ± 3	11 ± 3	9 ± 3	−2 (18)	**
Body composition measurement						
Body mass	(kg)	101 ± 25	102 ± 27	97 ± 26	−5 (5)	
Body fat	(%)	38 ± 8	38 ± 7	36 ± 8	−2 (5)	
Muscle mass	(kg)	34 ± 9	35 ± 9	34 ± 9	−1 (3)	

MVIC = maximum voluntary isometric contraction. GL = Gastrocnemius lateralis. GM = Gastrocnemius medialis. TA = Tibialis anterior. EMG_GL/TA_ = Gastrocnemius lateralis and tibialis anterior activation ratio. EMG_GM/TA_ = Gastrocnemius medialis and tibialis anterior activation ratio. EMG_TA/GL_ = Tibialis anterior and gastrocnemius lateralis activation ratio. EMG_TA/GM_ = Tibialis anterior and gastrocnemius medialis activation ratio. ROM = Range of motion. TUG = Timed up and go. Δ% = Percent change from Test 2 to Test 3. ^#^ = Difference between Test 2 and Test 3. * Significand difference between Test 2 and Test 3 (*p* ˂ 0.05). ** Significant difference between Test 2 and Test 3 (*p* ˂ 0.005).

## Data Availability

Data is not available due to restrictions privacy. The data presented in this study are available on request from the corresponding author.

## Supplementary Materials

The following supporting information can be downloaded at: https://www.mdpi.com/article/10.3390/ijerph20075413/s1, Table S1: Sand training exercises.
