# Supplementary material for: The Effects of a 12-Week-Long Sand Exercise Training Program on Neuromechanical and Functional Parameters in Type II Diabetic Patients with Neuropathy
Source: Int J Environ Res Public Health. 2023 Apr 5;20(7):5413. doi: 10.3390/ijerph20075413 (PMC10094138; doi:10.3390/ijerph20075413)
Supplement: Supplementary file 1 [file ijerph-20-05413-s001.zip › Table S1. Sand training exercises.pdf]

| TRAINING PERIOD | WEEK 1-4 | WEEK 5-8 | WEEK 9-12 |
| --- | --- | --- | --- |
| SESSION DURATION | 30 MINUTES | 37 MINUTES | 45 MINUTES |
| MAIN PART EXERCISES | 1. Jogging 16x<br>2. With the dorsal side of the toes, flicking sand 6xleft foot, 6xright foot<br>3. Caterpillar movements forward and backward 3xleft foot, 3xright foot<br>4. Dorsal flexion alternating 16x, both 8x<br>5. Heel rise 10x<br>6. With the dorsal side of the foot lifting sand and putting it to side 6xleft, 6xright<br>7. Sand grip with toes, bent leg lift, after knee extension, spreading the sand 3xleft, 3xright<br>8. Drawing palm tree, outward with the dorsal, inward with the plantar side of the foot 2xleft, 2xright<br>9. Covering foot with sand 1xleft, 1xright<br>10. walking in circle | 1. Jogging 24x<br>2. With the dorsal side of the toes, flicking sand 8xleft foot, 8x right foot<br>3. Caterpillar movements forward and backward 4xleft foot, 4xright foot<br>4. Dorsal flexion alternating 20x, both 12x<br>5. Heel rise 15x<br>6. With the dorsal side of the foot lifting sand and putting it to side 8xleft, 8xright<br>7. Sand grip with toes, bent leg lift, after knee extension, spreading the sand 4xleft, 4xright<br>8. Drawing palm tree, outward with the dorsal, inward with the plantar side of the foot 3xleft, 3xright<br>9. Covering foot with sand 1xleft, 1xright<br>10. walking in circle<br>11. walking on toes ¼ circle<br>12. walking on heel ¼ circle<br>13. walking on outer sole ¼ circle<br>14. walking on inner sole ¼ circle<br>15. walking in circle | 1. Jogging 32x<br>2. With the dorsal side of the toes, flicking sand 10xleft foot, 10xright foot<br>3. Caterpillar movements forward and backward 5xleft foot, 5xright foot<br>4. Dorsal flexion alternating 24x, both 16x<br>5. Heel rise 20x<br>6. With the dorsal side of the foot lifting sand and putting it to side 10xleft, 10xright<br>7. Sand grip with toes, bent leg lift, after knee extension, spreading the sand 5xleft, 5xright<br>8. Drawing palm tree, outward with the dorsal, inward with the plantar side of the foot 4xleft, 4xright<br>9. Covering foot with sand 1xleft, 1xright<br>10. With bending the support leg, pushing sand backward with the plantar side of the foot, pulling sand forward with the dorsal side of the foot 5xleft, 5x right<br>11. squat 8x<br>12. squat with rising onto the toes 8x<br>13. walking in circle<br>14. walking on toes ¼ circle<br>15. walking on heel ¼ circle<br>16. walking on outer sole ¼ circle<br>17. walking on inner sole ¼ circle<br>18. walking in circle with long strides<br>19. walking in circle |
